# Functional Assessment of Peroneal Muscles Using Ultrasound Imaging in Chronic Ankle Instability

**DOI:** 10.1002/jfa2.70088

**Published:** 2025-11-17

**Authors:** Abbis Jaffri

**Affiliations:** ^1^ Department of Physical Therapy Creighton University Omaha Nebraska USA

**Keywords:** muscle activation, rehabilitation, weight‐bearing

## Abstract

**Background:**

Lateral ankle sprains (LAS) result in chronic ankle instability (CAI), causing ongoing instability. Although peroneal muscle weakness is documented in CAI, surface electromyography shows similar activation patterns between CAI and healthy individuals, suggesting structural rather than neural deficits. Ultrasound imaging (USI) uniquely enables noninvasive assessment of muscle morphology and quality through cross‐sectional area and echogenicity measurements. However, previous USI studies examined peroneals only in nonweight‐bearing positions, potentially missing functional deficits. This study examines peroneal muscle characteristics in CAI versus healthy individuals specifically during weight‐bearing functional positions using USI.

**Methods:**

A case–control study was conducted with 58 participants (29 CAI and 29 healthy controls), aged 18–30 years. Cross‐sectional area (CSA), echogenicity (grayscale analysis where higher values indicate fatty infiltration/fibrosis), and functional activation ratio (FAR) of the peroneal muscles were assessed using USI in nonweight‐bearing (side lying) and weight‐bearing (bilateral‐leg standing (BLS) and single‐leg standing (SLS)) positions. CSA images were averaged from three measurements for each position.

**Results:**

The CAI group had significantly smaller CSA in BLS (*p* < 0.01) and SLS (*p* < 0.01) but not lying (*p* = 0.06), higher echogenicity indicating poorer muscle quality (69.7 ± 10.3 vs. 61.3 ± 7.0, *p* < 0.01), and lower FAR in both BLS (0.99 ± 0.13 vs. 1.13 ± 0.16, *p* < 0.01) and SLS (1.01 ± 0.17 vs. 1.12 ± 0.22, *p* = 0.03) compared to healthy controls.

**Conclusion:**

Individuals with CAI showed reduced peroneal muscle CSA, lower activation, and poorer muscle quality specifically in weight‐bearing positions compared to healthy controls. These findings suggest altered muscle function in CAI especially in functional weight‐bearing positions. This demonstrates the need to assess peroneals in functional weight‐bearing position compared to resting.

**Abbreviations**
BLS: bilateral standing
CAI: chronic ankle instability
CSA: cross‐sectional area
FAAM: foot and ankle ability measure
FAR: functional activation ratio
IdFAI: identification of Functional Ankle Instability
LAS: lateral ankle sprain
PFP: patellofemoral pain
SLS: single leg standing
USI: ultrasound imaging

## 1 Introduction

Lateral ankle sprains (LASs) are considered to be most common orthopedic injury [1]. However, a significant number, up to 73% [2], experience reinjury, with previous LAS being the top risk factor for further sprains [3]. Some individuals, known as “copers,” recover from a LAS and resume activities without lasting issues [4]. However, almost 70% of the individuals who suffer from an ankle sprain go on to develop a long‐lasting condition called chronic ankle instability (CAI) [5]. CAI is characterized by ongoing feelings of instability or episodes of the ankle “giving way” more than a year after the initial sprain [6].

After a LAS, sensorimotor functions often change, with noted strength deficits in the peroneal muscles (longus and brevis together) among those with LAS history [7, 8, 9]. A previous meta‐analysis examined 8 studies (*n* = 286 total) and found consistent eversion strength deficits of 10%–15% in CAI subjects measured via isokinetic dynamometry [7]. However, Donnelly et al. (2017) found this strength deficits did not correlate with surface EMG amplitude during functional tasks, suggesting structural rather than neural impairments [8]. Additionally, it is reported that there are similar motor unit recruitments between healthy and CAI subjects, however, still CAI subjects are unable to produce more force [10]. Considering the similar EMG profiles, but differences in force production, there appears to be other aspects of muscle function that could contribute to this deficit, such as alterations in muscle architecture that are not captured by surface EMG measurements.

We postulate that ultrasound imaging (USI) may be more useful in this group of patients, in terms of looking at muscle fibers length and the cross‐sectional area (CSA; the area of muscle measured perpendicular to its longitudinal axis) which can tell us about muscular adaptations. USI reliability for peroneal CSA has been established with excellent intrarater ICC values of 0.95–0.99 [11]. USI provides a noninvasive way to assess muscle structure including muscle size by measuring CSA and muscle quality by performing echogenicity analysis (the grayscale pixel intensity indicating muscle composition, where higher values suggest greater intramuscular fat and connective tissue) [12]. Additionally, USI can also be used to understand the muscle activation by taking functional activation ratios [13]. Functional activation ratio (FAR) can be determined by adjusting CSA in functional activity, such as weight‐bearing position relative to its size when at rest or nonweight‐bearing position, to show how much the muscle is activated beyond its baseline or quiet state [10, 14]. In contrast, surface electromyography (EMG), although common in research, has limitations, such as crosstalk and discomfort, and is not as readily available for clinical use [12]. The USI characteristics, such as muscle morphology, quality, and activation ratios, can explain the changes in force measures found when there were no significant changes in the EMG measures [10].

Although USI has been used sparingly in LAS research, one study showed smaller muscle sizes in those with LAS whereas the other study did not show any differences between CAI and healthy [12]. Previous USI studies in CAI showed conflicting results: Lobo et al. [15] found 23% smaller peroneal CSA in LAS subjects (*n* = 41) using standardized probe placement, whereas Abdeen et al. [16] found no differences but only assessed muscles at rest in nonweight‐bearing positions (*n* = 30). The mixed results in previous studies can be attributed to performing these measurements all in nonweight‐bearing positions which is not a functional position of peroneal muscles. To our knowledge, no prior research has examined the size, quality, and activity of the peroneal muscles in individuals with CAI while in a weight‐bearing functional position. The aim of this study is to compare the muscle size, quality, and activity of the peroneal muscles in this position between a group with CAI and a group of healthy controls in functional weight‐bearing position. We predict that the CAI group will show different behavior in the weight‐bearing position, specifically with reduced activation of the peroneal muscles compared to the healthy controls. We hypothesize that (1) CAI subjects will demonstrate smaller peroneal CSA specifically in weight‐bearing positions; (2) echogenicity will be higher indicating poorer muscle quality; and (3) FAR will be lower indicating reduced functional activation compared to healthy controls.

## 2 Materials and Methods

This case–control study was exploratory in nature with convenience sampling technique utilized and hence did not include a priori sample size calculation. The primary outcomes were peroneal muscle CSA, which reflect structural and functional deficits in CAI.

The independent variables were groups (CAI and healthy) and positions (nonweight‐bearing side lying and weight‐bearing bilateral‐leg standing (BLS) and single‐leg standing (SLS) position). The dependent variables were peroneal size (cross‐sectional area (CSA) of peroneal muscles), muscle quality (echogenicity), and functional activation ratio (FAR) assessed using ultrasound imaging (USI).

### 2.1 Participants

Fifty‐eight participants (29 CAI and 29 healthy matched controls) aged between 18 and 30 was included in this study. Participants were matched based on age, height, weight, and gender. Demographic information is outlined in Table 1. Participants were included in the “healthy” group if they had no history of lower extremity surgery, no history of ankle sprains, no lower extremity injury in the 6 months prior to enrollment, and no known neurological dysfunction. For those with CAI, additional inclusion criteria were based on previous literature [17]. Participants with CAI were included if they had a history of at least one significant ankle sprain, persistent symptoms for more than 3 months, and reported episodes of “giving way” or feelings of instability during activities such as walking, running, or standing on one leg. The inclusion criteria adhered to the guidelines set by the International Ankle Consortium [17]. All participants had experienced a significant lateral ankle sprain at least 12 months before joining the study, reported functional limitations (with a score of 85% or less on the Foot and Ankle Ability Measure (FAAM) Sport subscale), and expressed feelings of instability (scoring more than 10 on the Identification of Functional Ankle Instability (IdFAI)). Exclusion criteria for participation in both groups encompassed any past incidents of lower limb injuries or fractures, balance issues, or neurological conditions.

**TABLE 1:** Participant demographics.

Variable	CAI (*n* = 29)	Healthy (*n* = 29)	*p*‐value
Sex	21F; 8M	21F; 8M	—
Age (yrs)	21.65 ± 3.37	20.75 ± 3.40	0.31
Height (cm)	170.00 ± 10.31	170.02 ± 9.28	0.98
Weight (kg)	70.31 ± 13.71	65.40 ± 13.32	0.18
FAAM‐sport %	67.99 ± 15.58	99.75 ± 1.32	< 0.001
FAAM‐ADL %	87.64 ± 8.12	100 ± 0	< 0.001

Abbreviations: cm, centimeter; FAAM, Foot and Ankle Ability Measure; FAAM‐ADL; idFAI, Identification of Functional Ankle Instability; kg, kilograms; yrs, years.

### 2.2 Procedure

**FIGURE 1:**
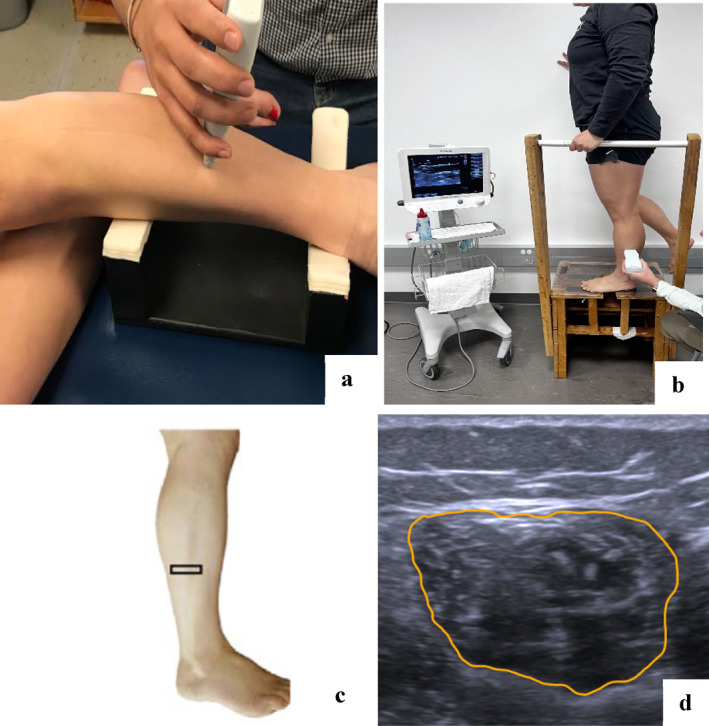
Ultrasound imaging. Ultrasound imaging was conducted in side‐lying, bilateral standing, and single leg standing positions. (a) Side lying position: This setup shows the peroneal muscles imaged in a nonweight‐bearing state in side‐lying position. (b) Single‐leg standing: This image captures the peroneal muscles during weight‐bearing in single leg standing. Similar position was use for bilateral standing with both feet planted with an equal distribution of weight on both limbs. (c) Probe placement: The ultrasound linear transducer is positioned at 50% of the fibular length, measured from the midpoint between the fibular head and the lateral malleolus. (d) Cross‐sectional area measurement: The cross‐sectional area (CSA) of the peroneal muscles is measured along the fascial borders indicated by a yellow line.

Institutional Review Board approval was secured for this project, and all participants provided written consent prior to data collection. After consenting and enrolling based on eligibility criteria, participants' demographics (age, weight, height, and duration of pain) were collected. Following demographic information, USI imaging was performed (Figure 1).

For USI, a Siemens Acuson Freestyle US system with a wireless 8‐MHz linear transducer (Siemens, Mountain View) was used. All the USI was performed at a depth of 3.5 cm with a gain of 11 and dynamic range of 70dB [10, 11, 12, 18]. These parameters, based on previous literature, were selected to optimize imaging of superficial muscles, such as peroneal, which lie within 2 cm of the skin surface [10, 11, 12, 18]. Notably, all ultrasound settings, such as gain and depth, were standardized and maintained consistently across all participants. The USI was performed in both nonweight‐bearing side lying position [12] (Figure 1a) and weight‐bearing BLS and SLS position (Figure 1b). The order of testing positions was randomized using a computer‐generated sequence to counter‐balance potential order effects across participants. Three images were taken and averaged for each position. Previously established reliable methods by Arellano et al. [18], Jaffri et al. [10], Koldenhoven et al. [12], and Angin et al. [11] were used for probe placement which was 50% of the fibular length (Figure 1c). Fifty percent of the fibular length was measured by using a measuring tape to calculate the total distance from the fibular head to the lateral malleolus and placing the marker at the 50% point [10, 11, 12, 18].

Participants stood on the leg that was scanned. In the healthy group, the right leg was scanned for everyone; in the CAI group, the leg on the same side as the self‐reported worst ankle was scanned. Three images were taken for peroneal muscles in both nonweight‐bearing side lying and the weight‐bearing BLS and SLS functional positions. A researcher with 5 years of expertise in USI conducted all the US evaluations. The compression from the ultrasound transducer was minimized to avoid altering muscle size. During weight‐bearing measurements, all participants were permitted to lightly touch a support bar for safety with standardized instructions to distribute weight equally between both limbs during BLS.

### 2.3 Data Processing

ImageJ version 1.50f, developed by the National Institutes of Health in Bethesda, Maryland, was employed for image analysis. All ultrasound images were deidentified and analyzed by a blinded investigator who had no knowledge of participant group allocation (CAI vs. control) or testing position until after all measurements were completed. All image analyses were performed by a single examiner with 5 years of USI experience. Muscle size and quality were evaluated from three images taken in each of two positions, with averages computed for each parameter. The cross‐sectional area (CSA) in cm^2^ was determined using ImageJ's freehand tool (Figure 1d). To normalize these size measurements and account for body size differences between participants, the CSA in cm^2^ was divided by the participant's body mass in kilograms, following established protocols in muscle morphology research [10, 19]. Additionally, FAR were computed for this study by dividing the CSA measured during a functional activity by the CSA measured at rest [14].

$$\text{Ratio}:\frac{\text{CSA}\left(\text{weight}‐\text{bearing}\;\text{position}\right)}{\text{CSA}\left(\text{nonweight}‐\text{bearing}\;\text{position}\right)}$$

Muscle echogenicity was calculated using grayscale analysis in ImageJ on the same ROI used for CSA, averaged across three images per position, which minimizes variability, measured on a 0‐to‐255 scale (black to white), where higher echogenicity indicates lower muscle quality.

### 2.4 Statistical Analysis

Data analysis was conducted with IBM Statistics software (version 26.0, SPSS Inc., Chicago, IL, USA). We evaluated the normality, skewness, and kurtosis of the dependent variables. The intraclass correlation coefficient (ICC) was employed across the groups for the three repeated CSA measurements taken to check the consistency of CSA measurements of the peroneal muscle images. Differences in demographic characteristics (age, weight, height, and pain duration) and patient‐reported outcomes (IdFAI and FAAM‐Sport) between groups were analyzed using an independent *t*‐test. For muscle size comparisons across groups and between nonweight‐bearing side lying and weight‐bearing BLS and SLS positions, a 2 × 2 repeated‐measures split‐plot ANOVA was applied. Independent *t*‐test were performed to understand pairwise differences for the post hoc analysis. The FAR were compared between groups with an independent *t*‐test. Statistical significance was set at an *α* level of less than 0.05. Effect sizes were calculated using Cohen's d with a 95% confidence interval (CI), classifying the strength of the effect as trivial (0–0.2), small (0.21–0.5), moderate (0.51–0.8), or large (> 0.8) [20].

## 3 Results

All dependent variables were found to be normally distributed, confirmed by evaluations of skewness, kurtosis, and Shapiro–Wilk test for normality (*p* > 0.05). There were no statistically significant differences (*p* > 0.05) in height, mass, or age between the two groups as detailed in Table 1. The reliability of peroneal muscle cross‐sectional area (CSA) measurements was excellent, with an intraclass correlation coefficient (ICC) ranging from 0.95 to 0.99.

**FIGURE 2:**
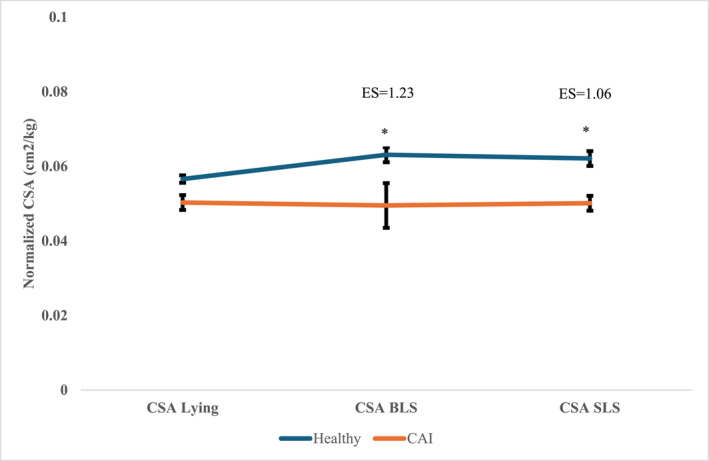
Interaction plot of Peroneal CSA in CAI and healthy individuals. Blue line represents the healthy group and CAI line represents the CAI group. * Denotes statistical significance. BLS: bilateral standing, CAI: chronic ankle instability, CSA: cross‐sectional area, ES: effect size, and SLS: single‐leg standing.

A significant interaction (*p* = 0.006) was noted between the groups (chronic ankle instability (CAI) versus healthy) and positions (nonweight‐bearing vs. weight‐bearing) for the peroneal muscle CSA (Figure 2). The CSA was smaller in the CAI group, significantly in the weight‐bearing single leg stance (BLS and SLS) position (see Figure 2). The effect sizes for CSA between groups demonstrated moderate to large differences, with the healthy group showing larger CSA values than the CAI group: d = 0.51 for the nonweight‐bearing lying position, with effect sizes increasing in weight‐bearing positions (*d* = 1.23 for BLS and d = 1.06 for SLS), indicating greater group differences when the peroneal muscles were functionally loaded. Post hoc pairwise analysis using independent *t*‐test revealed significant differences between CAI and healthy BLS (*p* < 0.01) and SLS (0.001) positions, but no differences were observed in nonweight‐bearing position (*p* = 0.052) (Table 2 and Figure 2). Additionally, muscle echogenicity was significantly higher (*p* < 0.01) in the CAI group compared to the control group, indicating poorer muscle quality in the CAI group compared to the control (Table 2). The FAR scores were significantly higher in the BLS healthy group compared to the CAI group (Table 2) as well as in the SLS healthy group compared to the CAI group (Table 2).

**TABLE 2:** Ultrasound imaging outcomes.

Variable	CAI (*n* = 29)	Healthy (*n* = 29)	*p*‐value	Cohen's d [95% CI]
Peroneal CSA (cm^2^/kg)				
Lying	0.050 ± 0.014	0.057 ± 0.011	0.06	0.51 [0.01, 1.03]
BLS	0.050 ± 0.013	0.063 ± 0.011	< 0.01	1.23 [0.68, 1.78]
SLS	0.050 ± 0.013	0.062 ± 0.011	< 0.01	1.06 [0.52, 1.60]
Functional activation ratio				
Far BLS	0.99 ± 0.13	1.13 ± 0.16	< 0.01	0.96 [0.43, 1.49]
Far SLS	1.01 ± 0.17	1.12 ± 0.22	0.03	0.56 [0.04, 1.08]
Muscle quality				
Echogenicity	69.70 ± 10.30	61.34 ± 7.01	< 0.01	0.95 [0.42, 1.48]

Abbreviations: BLS, bilateral‐leg standing; CAI, chronic ankle instability; CSA, cross‐sectional area; FAR, functional activation ratio; SLS, single‐leg standing.

## 4 Discussion

To our knowledge, this is the first study to investigate morphological changes in peroneal muscles in both nonweight‐bearing and weight‐bearing positions in the CAI group (Table 2 and Figure 2). Our key findings demonstrate that individuals with CAI exhibit: (1) smaller peroneal CSA specifically in weight‐bearing positions (Table 2), (2) higher echogenicity indicating poorer muscle quality, and (3) lower FARs (Table 2). These findings align with Lobo et al. [15] who found 23% smaller peroneal CSA in LAS subjects but extend their work by demonstrating that differences are magnified during functional weight‐bearing tasks. This contrasts with Abdeen et al. [16] who found no CSA differences, likely because they assessed muscles only at rest in nonweight‐bearing positions.

Notably, Lobo et al. [15] showed significant differences in the CSA of peroneal muscles between individuals with LAS and a healthy control group, with the peroneal muscles being significantly smaller in those with LAS. Although we also noted a reduced size in the peroneal muscle group, our study focused on CAI rather than LAS, suggesting that these muscle deficits endure over time. This is crucial because it provides another explanation for the common CAI symptom known as “giving way” [6]. This symptom indicates that weakness in the primary lateral muscular wall might be a key factor in the perception of ankle instability, which in turn heightens the risk of reinjury [7, 8]. In contrast, Abdeen et al. [16] found no size differences in peroneal muscles among healthy individuals, copers, and those with CAI. However, their study did not explore neuromuscular control since the muscles were measured in a resting state, where they are not actively engaged. Thus, even if muscle size appears similar, there might be significant differences in how these muscles are controlled and activated between the groups [16]. This explains the recommendations made by Donnelly et al., [8] who highlighted the functional differences of the peroneal muscles between weight‐bearing and nonweight‐bearing positions, suggesting that these muscles should be tested in the weight‐bearing functional position.

We also observed that the peroneal muscles in the CAI group exhibited higher echogenicity compared to those in the healthy group, indicating poorer muscle quality. Higher echogenicity is typically associated with fatty infiltration and fibrosis, which can impair muscle repair and regeneration [21, 22]. The increased amount of adipose tissue within the muscle leads to greater brightness in ultrasound images due to the difference in acoustic impedance between muscle and adipose tissue [21, 22]. Furthermore, the echo‐intensity of the muscle correlates with muscle strength and the presence of adipose tissue [21, 22]. Sakai et al. [23] reported increased echogenicity in the peroneal muscles of CAI subjects compared to the non‐CAI side, yet they found no difference in CSA of the peroneal muscles. This discrepancy suggests that orthopedic conditions might degrade muscle quality more than muscle quantity [23]. It is plausible that reflex inhibition and reduced muscle activity postsprain contribute to the accumulation of adipose tissue within the muscle [23]. Rehabilitation, particularly through resistance training, improves both muscle quality and quantity [12]. We aimed to investigate whether there are changes in the size and quality of the peroneal muscles following rehabilitation. Similarly, our muscle quality changes are also novel because we compared our group to the matched health control which is a truer comparison as compared to within the subject design since the individuals with CAI have shown to reduce their physical activity which can change the muscle characteristics in both limbs and may not provide a truer comparison. Additionally, unlike our study, Sakai et al. [23] did not compare the CAI group with a healthy control group, which might explain why they did not observe changes in muscle size between the two limbs. This comparison is more accurate than a within‐subject design since individuals with CAI tend to reduce their physical activity, which can alter muscle characteristics in both limbs, potentially skewing the comparison.

Similar to our FAR results in this study, impairments in neuromuscular recruitment patterns of peroneal muscles have also been shown in the past. Decreased reaction times have been reported in the peroneal muscle group in CAI group [24]. However, the activation measured through EMG studies has mixed findings with some studies finding impairments in muscle activation whereas others did not find changes in muscle activation [12]. We believe that USI, although different than EMG signal processing, can still be helpful in understanding activation of the muscle in different functional activities since we can visualize the CSA without any fear of cross‐talk from other muscles which can help us accurately developing activation ratios [10, 14, 25]. We utilized simple weight‐bearing positions (BLS and SLS) in this study, which could be further expanded to include other functional activities in future research, for example, understanding the activity of these muscles during heel raises using USI. Although we did not use EMG, our approach captured functional activation indirectly through FAR, which reflects morphological changes during loading [13]. Previous studies have validated ultrasound‐based activation ratios as a surrogate for muscle activation in functional tasks [13]. Additionally common method, such as EMG, to measure activation of muscles has notable limitations, including signal contamination from crosstalk, which complicates interpretation during functional tasks [13]. In contrast, our ultrasound‐based approach circumvents these issues, eliminating crosstalk and reducing participant discomfort, while offering a clinically practical means to assess muscle engagement [13]. This morphological approach to assessing neuromuscular function complements existing EMG studies by capturing structural adaptations that occur with functional loading, which EMG alone cannot detect. The function of the peroneal muscles becomes more significant in weight‐bearing contexts because the peroneus longus depresses the head of the first metatarsal due to its strong pull at its insertion point [26]. It maintains the transverse arch of the foot due to its cross‐foot orientation, steadies the leg on the foot by pulling laterally, and prevents medial collapse. The musculotendinous structure of the peroneals primarily provides lateral stability [26]. There is a notable difference in the function of the peroneal muscles (especially the peroneus longus) between nonweight‐bearing and weight‐bearing positions. During weight‐bearing, the peroneus longus stabilizes the first ray, particularly during the stance phase [27]. This functional activation of the peroneus longus might not be observed in non‐weight‐bearing positions, potentially missing out on crucial information [28]. It has been shown in the literature that there is difference in the activation of peroneus longus in weight bearing conditions. The reasoning for that can be.

1. Stabilizing the forefoot on the weight bearing condition (you have both single leg and double leg weight bearing for your Ultrasound study) during dynamic activities [26].
2. It is also possible that tactile stimulation is also facilitating the functional activation of the peroneal muscles that is not possible in the nonweight‐bearing condition [26].

This holds significant clinical implications for the management of individuals with CAI. Our findings demonstrate the importance of assessing muscles in functional positions rather than relying solely on traditional nonweight‐bearing assessments. The weight‐bearing‐specific deficits we identified suggest that rehabilitation programs should emphasize exercises in loaded positions to address these functional impairments. Further studies integrating dynamic ultrasound imaging with simultaneous EMG and strength measures would provide more comprehensive understanding of the neuromuscular adaptations in CAI.

### 4.1 Limitations

This study has its limitations. We captured images of the peroneal muscles in the most functional BLS and SLS position to evaluate them in a loaded state with the stance foot on the ground. Nonetheless, the FAR measures still need further refinement in USI research, especially for lower extremity studies. It is essential to validate the FAR in lower limb muscles using concurrent EMG studies in different functional positions. Future investigations should consider these muscles during other functional activities or positions as USI uniquely allows for assessment of the peroneal muscles in both nonweight‐bearing and weight‐bearing functional scenarios. Furthermore, analyzing other extrinsic lower limb muscles, including those on the medial and lateral leg, can offer a fuller picture of distal muscle changes in the CAI group. This being a cross‐sectional study, it does not prove causation. Additionally, although smaller CSA may contribute to reduced force‐generating capacity, we did not directly measure strength; therefore, this interpretation should be considered speculative. We did not look at correlations with patient‐reported outcomes, thus it is uncertain if these changes relate to pain or dysfunction. The images were static, leaving it unclear how these findings might apply to dynamic function. Lastly, since we used a convenience sample and our study was exploring, we did not perform a power analysis to determine the sample size. This means the findings may not be generalizable to larger populations. Future studies can use the variances from our research to help determine an appropriate sample size.

## 5 Conclusion

This study is the first to examine the peroneal muscle group in the weight‐bearing functional position in individuals with CAI. The results indicated that those with CAI had a smaller peroneal muscle CSA in the weight‐bearing position, poor muscle quality, and lower FAR compared to a healthy group. Future research is required to explore how muscle size and activation ratios change due to rehabilitation in this population. Additionally, future research should integrate these measures and dynamic imaging (e.g., cineloops) to further validate neuromuscular control assessment.

## Author Contributions

A.J. collected and analyzed the data. The author designed the study, drafted the manuscript, and critically revised the manuscript for important intellectual context and wrote the final version of this manuscript.

## Ethics Statement

The study was approved by the Institutional Review Board of University of Virginia.

## Consent

The author has nothing to report.

## Conflicts of Interest

The author declares no conflicts of interest.

## Data Availability

Data and materials can be available upon request.
